# Artificial intelligence for non-invasive glycaemic-events detection via ECG in a paediatric population: study protocol

**DOI:** 10.1007/s12553-022-00719-x

**Published:** 2023-01-23

**Authors:** Martina Andellini, Salman Haleem, Massimiliano Angelini, Matteo Ritrovato, Riccardo Schiaffini, Ernesto Iadanza, Leandro Pecchia

**Affiliations:** 1 School of Engineering, University of Warwick, CV4 7AL Coventry, UK; 2 HTA Unit, Bambino Gesù Children’s Hospital, IRCCS, Rome, Italy; 3 HTA Research Unit, Bambino Gesù Children’s Hospital, IRCCS, Rome, Italy; 4 Diabetes Unit, Bambino Gesù Children’s Hospital, IRCCS, Rome, Italy; 5 Medical Biotechnologies Department, University of Siena, Siena, Toscana, Italy

**Keywords:** Artificial Intelligence (AI), Non-invasive glycaemia detection, ECG signal processing, paediatric type 1 diabetes (T1D)

## Abstract

**Purpose:**

Paediatric Type 1 Diabetes (T1D) patients are at greater risk for developing severe hypo and hyperglycaemic events due to poor glycaemic control. To reduce the risk of adverse events, patients need to achieve the best possible glycaemic management through frequent blood glucose monitoring with finger prick or Continuous Glucose Monitoring (CGM) systems. However, several non-invasive techniques have been proposed aiming at exploiting changes in physiological parameters based on glucose levels. The overall objective of this study is to validate an artificial intelligence (AI) based algorithm to detect glycaemic events using ECG signals collected through non-invasive device.

**Methods:**

This study will enrol T1D paediatric participants who already use CGM. Participants will wear an additional non-invasive wearable device for recording physiological data and respiratory rate. Glycaemic measurements driven through ECG variables are the main outcomes. Data collected will be used to design, develop and validate the personalised and generalized classifiers based on a deep learning (DL) AI algorithm, able to automatically detect hypoglycaemic events by using few ECG heartbeats recorded with wearable devices.

**Results:**

Data collection is expected to be completed approximately by June 2023. It is expected that sufficient data will be collected to develop and validate the AI algorithm.

**Conclusion:**

This is a validation study that will perform additional tests on a larger diabetes sample population to validate the previous pilot results that were based on four healthy adults, providing evidence on the reliability of the AI algorithm in detecting glycaemic events in paediatric diabetic patients in free-living conditions.

**Trial registration:**

ClinicalTrials.gov identifier: NCT03936634. Registered on 11 March 2022, retrospectively registered, https://www.clinicaltrials.gov/ct2/show/NCT05278143?titles=AI+for+Glycemic+Events+Detection+Via+ECG+in+a+Pediatric+Population&draw=2&rank=1.

**Supplementary information:**

The online version contains supplementary material available at 10.1007/s12553-022-00719-x.

## Introduction

### Background

Type 1 diabetes mellitus (T1D) has been one of the most common chronic diseases among children and adolescents since the last two decades. The prevalence of T1D worldwide varies significantly across countries [1, 2]. The European Childhood Diabetes Registry shows a 3.4% increase per annum in incidence rate in Europe suggesting that the incidence rate will duplicate over the next 20 years [3]. The paediatric patients suffering from T1D are at greater risk for developing acute rather than chronic complications, compared to adult patients [4]. In fact, chronic complications usually appear after decades of T1D, and it is extremely uncommon for children or adolescents to develop significant diabetic microvascular or macro-vascular complications. Therefore, it is pivotal that those paediatric patients achieve an optimal T1D management by reducing the HbA1c levels and glycaemic variability.

Several studies confirmed that a good management of diabetes during the paediatric age not only reduces the risk of adverse events but is able to delay the onset of long-term complications [5, 6]. The T1D management requires daily Self-Monitoring of Blood Glucose (SMBG). The increase in daily frequency of SMBG is highly correlated with the reduction in HbA1c level which is associated with occurrence of complications [7]. However, paediatric and adolescent patients may struggle with SMBG and self T1D management not only due to paradigm shift in diet, but also because SMBG require the use of finger pricks which are invasive and cumbersome. This, as a consequence, may affect patient compliance with the glucose measurement [8]. As an alternative, Continuous Glucose Monitoring (CGM) can infer glucose levels in real-time based on glucose in interstitial fluid which may help in reducing self-care burden in terms of improvement in non-invasive capture of glycaemic values per day without frequent finger prick. The use of CGM devices has shown significant improvement of glucose control among diabetic patients [9, 10]. Nevertheless, CGM devices are expensive and can be only worn between 7 and 14 days [11, 12]. Besides, the reliability and accuracy of the CGM values during low blood glucose levels has been questioned in some studies as there are no international standards for regulating the measurements [13–17]. They are not designed to combine glycaemic values with other physiological parameters, food intake or activity measures, which are pivotal in diabetes management. Above all, the CGM devices require cannula to be inserted in subcutaneous tissue which still make them invasive and intolerable by the skin of the paediatric subjects.

Over the last few years, with increased number of wearable sensors developed for tracking physiological signal, several studies have been proposed to estimate the blood glucose levels by combining CGM data with physiological signals for determining glycaemic events [18, 19]. These strategies have the potential to overcome the limitation of the CGM devices in terms of non-invasive detection blood glucose levels [20–23], while combining physiological parameters, physical activity, and food intake for estimation of glycaemic events such as hypoglycaemia (event with blood glucose lower than normal) and hyperglycaemia (event with blood glucose higher than normal) [20, 24, 25]. Therefore, the development of a non-invasive real-time monitoring system which is able to estimate blood glucose levels and/or detect glycaemic events is of great interest.

Previous clinical studies have shown that glycaemic events affect certain cardiac characteristics of the heart in healthy, type 1 diabetic and type 2 diabetic subjects [5, 26–30]. These cardiac characteristics can be represented by Heart Rate Variability (HRV) parameters such as beat intervals, power spectrum etc. [31]. Moreover, also the relationships of heart rate with hyperglycaemia has been studied, emerging that it was associated with reduced HRV [32]. The recurring electrocardiogram (ECG) alterations when hypo or hyperglycaemic event occurs are P-R interval shortening, ST-segment depression, T-wave flattening and QT-interval prolongation during hypoglycaemic events, reduction of R-R variability, QT interval variability, corrected QT interval variability and increase of P-R interval [33, 34].

With the relatively recent introduction of Artificial Intelligence (AI) in health diagnosis and monitoring, several machine learning models have been developed for hypoglycaemia and hyperglycaemia detection, all relying on the analysis of the previously mentioned ECG alterations. A literature review highlighted that much more efforts have been spent on the study of hypoglycaemia classification and detection compared to hyperglycaemia (39 studies vs. 5, respectively) [35]. Results from our preliminary study, carried out in 2019, are promising in this field [18, 19]. In these studies, we successfully applied advanced deep learning and mathematical modelling techniques, coupled with raw ECG signals, to identify nocturnal glycaemic episodes with a high degree of accuracy. However, this initial pilot work was conducted on healthy adults, thus, without a clinical classification of diabetes. For this reason, this study proposes to expand such results to a wider cohort of paediatric diabetes patients, creating a dedicated, and extended dataset specific to enhance the accuracy, robustness and validation of machine learning and deep learning models. It will lead to the development of the generalized model for non-invasive detection of glycaemic events based on ECG signals.

### Rationale

This study is a validation and developmental project. We have conducted a pilot study showing that hypoglycaemic events can be automatically detected using short-term ECG signals recorded with wearable devices in free-living conditions by developing deep learning based models under personalized setup [18, 19]. The proposed system can automatically learn patterns in the ECG heartbeat, discriminating between heartbeats recorded during low or normal glucose levels in the same subject. The deep learning approach has shown significantly better performance compared to traditional machine learning due to their capability of extracting ECG heartbeat patterns at both spatial and temporal levels. However, we need to demonstrate the accuracy, robustness, and generalization of the AI model for detecting glycaemic events in diabetic patient groups. As a result, the proposal is focused on gathering physiological data from diabetes patients in order to test and validate our AI models.

### Objectives

The purpose of this single-centre, observational, single-arm study is to validate our novel AI model for non-invasive detection of glycaemic events. To achieve this, we aim to detect and measure the relationship between glucose fluctuations in the blood and ECG variability under hypoglycaemic, glycaemic and hyperglycaemic events. The ECG signals will be recorded via non-invasive wearable devices for physiological signal measurements whereas blood glucose will be monitored via CGM for T1D paediatric patients under free-living conditions.

## Materials and methods

### Study setting

This is a single-centre, observational, single-arm study. It is conducted at the Unit of Endocrinology and Diabetes in Bambino Gesù Children’s Hospital, Rome, Italy. The study adheres the ethical principles stated in the Declaration of Helsinki and the National Health and Medical Research Council statement. The protocol was defined following the SPIRIT 2013 checklist (recommended items to address in a clinical trial protocol and related documents) the study has been prospectively registered in clinicaltrial.gov (ClinicalTrials.gov identifier: NCT05278143).

### Inclusion and exclusion criteria

Both male and female subjects diagnosed with T1D, aged less than 18 years old who are currently under the care of the Unit of Endocrinology and Diabetes of Bambino Gesù Children’s Hospital, Rome, Italy and who use continuous glucose monitoring (CGM) systems are eligible to be involved in the study. Patients with coexistence of celiac disease or non-diabetic hypoglycaemia or cardiovascular pathologies and cardiac arrhythmias are excluded. Furthermore, patients who are pregnant or becoming pregnant during the study are also excluded as well as patients that use standard finger prick glucometer to measure glycaemic values.

Inclusion and exclusion criteria are summarized in Table 1.

**Table 1:** Inclusion and Exclusion Criteria

**Inclusion criteria**
Age less than 18 years old
Diagnosed with type 1 diabetes
Use of continuous glucose monitoring systems (CGM)
**Exclusion criteria**
Use of standard finger prick glucometer to measure glycaemic values
Being pregnant or becoming pregnant during the study
Coexistence of celiac disease
Coexistence of non-diabetic hypoglycaemia
Coexistence of cardiovascular pathologies and cardiac arrhythmias

### Enrolment procedure

Written, informed consent is obtained from parents or caregivers of each paediatric patient who decided to join the study during regular visits. Paediatric patients and parents or caregivers are trained at the end of the regular diabetes visit by a member of the study team. During the study, participants continue to receive the diabetes care which they manage regularly as per clinical advice. In this way, the study participation will not affect in any way the clinical path of the recruited patients. Participants will be assigned a code number. The database that links the subject identity to the code number will be kept by the medical doctor of the study team and protected by username and password.

### Sample size

In this study, 64 paediatric patients with T1D are going to be enrolled. The number of subjects was chosen to ensure that there is sufficient evidence that the approaches we propose are generalizable and consistent with prior similar research that enrolled between 21 and 43 subjects In this study, 64 paediatric patients with T1D are going to be enrolled. The number of subjects was chosen to ensure that there is sufficient evidence that the approaches we propose are generalizable and consistent with prior similar research that enrolled between 21 and 43 subjects [36, 37].

### Study procedure

A graphical representation of the study protocol is showed in `. 1. As per inclusion criteria, the study participants continue to use their CGM device they are already using. During their routine diabetes hospital visit, the participants are asked to wear an additional wearable device, Medtronic Zephyr BioPatch, for recording the physiological data for a period of up to three days. After receiving the training session and relevant information about the study, the participants are allowed to return home with the wearable device attached. During the hospital visit, the quality-of-life questionnaire for paediatric patients (PEdsQL) is submitted to recruited patients [38, 39]. They are asked to answer questions on how T1D affects their daily activities.

During the monitoring days, patients can continue their daily activities undisturbed, without any changes in either physical activities or diet. In this way, data gathered from free-living conditions are obtained. Patients receive regular contact from the research team not only to check on their safety and wellbeing, but also to ensure the data collection is successful. At the end of the third day, patients should return the devices to the hospital.

**Fig. 1:**
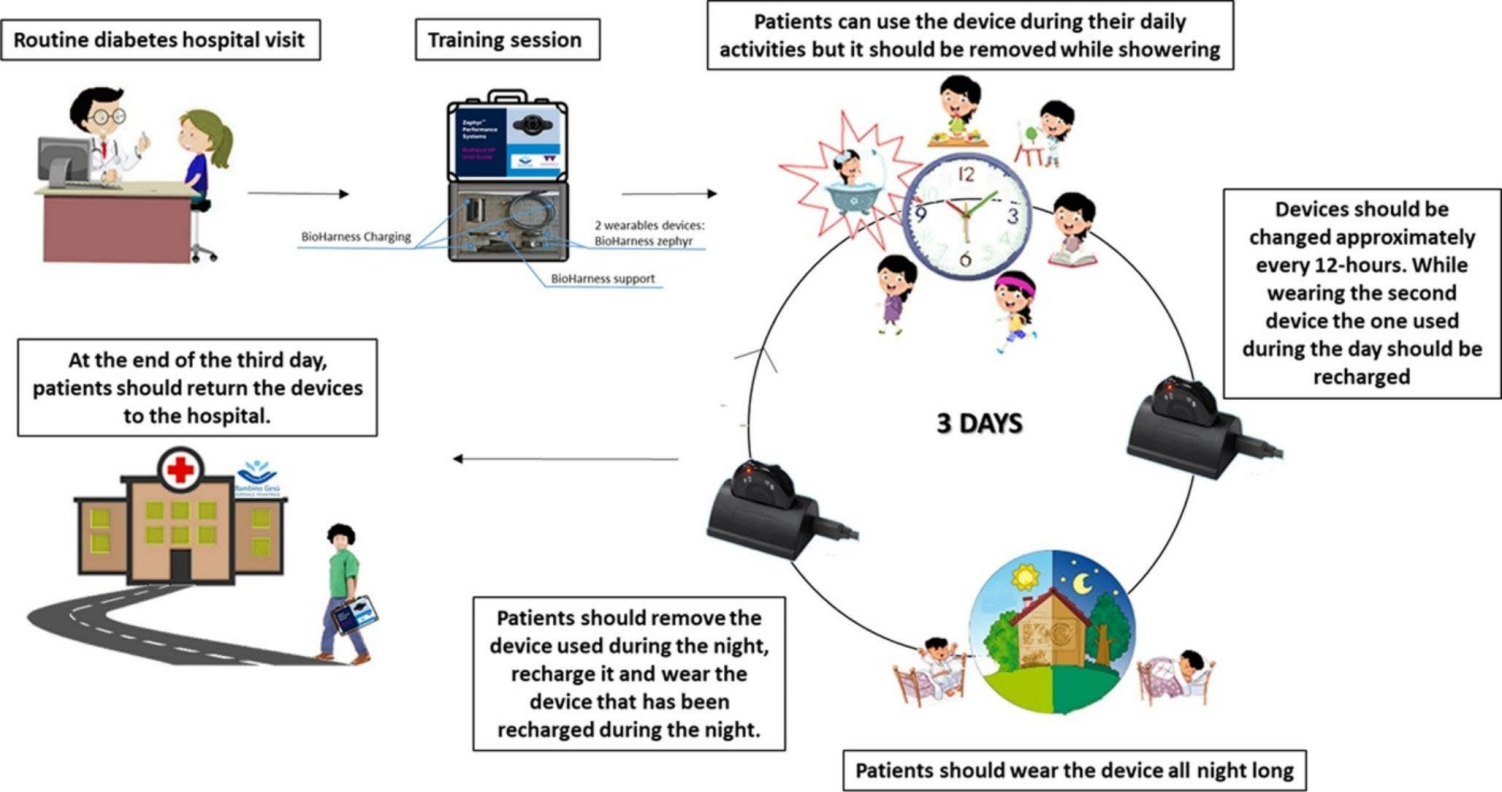
Shows the study procedure. During the patients’ routine diabetes hospital visit, recruited patients (that already use CGM sensors) are asked to wear an additional wearable device, Medtronic Zephyr BioPatch, for recording the physiological data for a period of up to 3 days. After receiving the training session and relevant information about the study, the participants are allowed to return home with the wearable device attached. During the hospital visit, the PEdsQL is submitted to recruited patients. During the monitoring days, patients can continue their daily activities undisturbed, without any changes in either physical activities or diet. They should wear the sensor during the day and the night and remove it while showering. The device should be approximately charged every 12-hours. For this reason, patients were provided with two devices. While wearing the second device the one used during the day should be recharged and vice versa. At the end of the third day, patients should return the devices to the hospital (This picture was created by the authors)

Continuous glucose levels are measured using the CGM device that each patient already use. Patients who use FreeStyle Libre Flash glucose monitoring system, Dexcom G6 or Medtronic Guardian sensor are included in the study. The former measures the interstitial glucose every 15 min, the others every five minutes. Each glucose sensor can be used for up to two weeks, also while showering, and, according to the manufacturer, it does not require any calibration with finger pricks (see Table 2).

**Table 2:** Main technical characteristics of Freestyle Libre, Dexcom G6 and Medtronic CGM sensors. [Adapted by ECRI Institute]

MANUFACTURER		Abbott Diabetes Care Div Abbott Laboratories Inc FreeStyle Libre Flash Glucose Monitoring System	Dexcom Inc G6 CGM System	Medtronic Diabetes USA Div Medtronic Inc Guardian Sensor 3 with Guardian Connect
**SAMPLE TYPE**		Interstitial fluid	Interstitial fluid	Interstitial fluid
**MEASUREMENT RANGE, mg/dL**		40–500	40–400	40–400
**SENSOR**				
	**Life, days**	≤ 14	≤ 10	≤ 7
	**Type**	Filament	Transcutaneous	Transcutaneous
	**Placement**	Back of upper arm	Abdomen (adult), abdomen and upper buttocks (paediatric)	Abdomen and arm
**TRANSMITTER**				
	**Water resistant**	Submerged ≤ 1 m (3 ft) and 30 min	Submerged ≤ 2.4 m (8 ft) and ≤ 24 h, IP28	IPX8
	**Operating time, hr**	348	3 months	170
**RECEIVER**				
	**Smartphone compatible**	Yes	Yes	Yes
	**Display type**	LCD	Not specified	NA (depends on phone)
	**Frequency of glucose readings shown**	Every min (stored every 15 min)	Every 5 min	Every 5 min
	**Rechargeable/replaceable Battery**	Rechargeable	Rechargeable	NA (depends on phone)
	**Life, days**	7	2	NA (depends on phone)
	**Memory: Number of stored readings with timestamp**	90 days of normal use including continuous glucose readings (stored every 15 min) and daily blood glucose results	30 days of data	90 days via Mobile App
**DATA MANAGEMENT**				
	**Software**	FreeStyle Libre	Dexcom Clarity (web-based diabetes management software, automatically uploads data when using a smart device)	CareLink Professional
	**Patient input**	Food, insulin, exercise, medication, ≤ 6 customizable notes; reminders, 3 predefined (check glucose, take insulin, alarm) and ≤ 9 reminders customizable through software	Carbohydrates, exercise, insulin, health events	Event markers for meals, insulin injections, exercise, blood glucose
**ALERT INDICATORS, TYPE**		Audible or vibrating	Audible, vibrating, visual	Audible or vibrating
**ALARMS**				
	**High/low glucose concentration**	No	Yes	Yes
	**Rate of change**	No	Yes	Yes
	**Predictive**	No	Yes	Yes

The Medtronic Zephyr BioPatch is a CE marked device and it works with 250 Hz sampling frequency and ECG amplitude between 0.25 and 15 mV. The performance of this sensor as a remote patient monitoring device was assessed and validated in previous clinical trials^1^. To acquire the biomedical signals, the wearable device is positioned using two gel electrodes on the patient’s chest, below the sternal notch. This device should be removed before bathing, showering, or swimming, and then worn again as explained during the training session. The device can store up to three days of ECG recordings, its battery can last for 36 h and can be fully charged in less than one hour. Therefore, each volunteer will be given two devices and instructed to change it approximately every 12-hours before showering. The Zephyr also records breathing waveform and 3-axis accelerations through which activity level and posture can be computed and physical activity can be assessed. Although the sensor can remotely record real time signals via Bluetooth, this functionality will be disabled, and the data will be downloaded by the researcher at the end of three to four days monitoring.

### Outcome measures

#### Primary outcomes

The primary outcome of the study is the detection of glycaemic events.

The glycaemic events can be determined non-invasively via ECG signals by the automated AI algorithm which are trained according to glucose measurements from the CGM and the cutoff-criteria for different glycaemic events: severe hypoglycaemia (glucose level < 50 mg/dl), hypoglycaemia (50 mg/dl < glucose level < 70 mg/dl), normal glycaemia (70 mg/dl < glucose level < 180 mg/dl), hyperglycaemia (180 mg/dl < glucose level < 240 mg/dl), severe hyperglycaemia (glucose level > 240 mg/dl).

The difference in ECG signals for different glycaemic events can be quantified through the assessment of the ECG variables (heart rate (BPM), physical activity and posture (lying, standing, walking, running) and Heart Rate Variability (HRV) features.[31] These features can be categorized as:

- Interval across different fiducial points (millisecond): We have five fiducial points (P.Q.R,S,T) and we can have total of 9 intervals among them.
- Slope across different fiducial points (mV/ms): We have five fiducial points (P.Q.R,S,T) and we can have total of 9 slopes among them.
- Absolute power (ms^2^/Hz): The signal energy can be determined for 5 min ECG excerpt within Ultra Low Frequency (ULF) (≤ 0.003 Hz), Very Low Frequency (VLF) (0.0033–0.04 Hz), Low Frequency (LF) (0.04–0.15 Hz) and High Frequency (HF) (0.15–0.4 Hz).

#### Secondary outcomes

Clinical outcomes will be assessed through the analysis of glycaemic values collected from CGM device. The condition of each patient is evaluated according to glycated haemoglobin level (HbA1c), mean glucose level and glycaemic variability, along with the frequency of glycaemic events (severe hypoglycaemia, hypoglycaemia, hyperglycaemia, and severe hyperglycaemia) and the time spent by subjects (minutes per day) in a specific range of glycaemic values (severe hypoglycaemia, hypoglycaemia, hyperglycaemia, and severe hyperglycaemia). Table 3 shows complete list of glycaemic variables with their measure’s units and cut-off criteria.

**Table 3:** Glycaemic Variables with their Measures/Units and Cut-off Criteria

Glycaemic variables	Measures-Unit	Cut off Criteria
Glycated haemoglobin level (HbA1c)	(%, mmol/mol)	-
Mean glucose level	mg/dL	-
Glycaemic variability	mg/dL	-
Frequency of severe hypoglycaemic events	Frequency (%)	Glucose level < 50 mg/dl
Time in severe hypoglycaemia	Min/day	Glucose level < 50 mg/dl
Frequency of hypoglycaemic events	Frequency (%)	50 mg/dl < Glucose level < 70 mg/dl
Time in hypoglycaemia	Min/day	50 mg/dl < Glucose level < 70 mg/dl
Normal time in range	Min/day	70 mg/dl < Glucose level < 180 mg/dl
Frequency of hyperglycaemic events	Frequency (%)	180 mg/dl > Glucose level > 240 mg/dl
Time in hyperglycaemia	Min/day	180 mg/dl > Glucose level > 240 mg/dl
Frequency of severe hyperglycaemic events	Frequency (%)	Glucose level > 240 mg/dl
Time in severe hyperglycaemia	Min/day	Glucose level > 240 mg/dl

Furthermore, the quality of life of recruited patients with T1D will be assessed through PedsQL questionnaire. The health-related quality of life questionnaire for paediatric age follows the World Health Organization (WHO) recommendation from 1948 for investigating the quality of life of children through the physical, psychological (emotional and cognitive aspects), and social health dimensions [38, 39].

### Data management

The data collected will be biomedical signals (including ECG, Breathing), continuous glucose levels, biomechanical variables (3D trunk accelerations), and personal information (age, gender). All study data will be anonymized and stored on the secure Bambino Gesù Children’s Hospital server. The data will be securely stored in a folder accessible only by researchers involved in the study with username and password.

### Data analysis and modelling

#### Data processing

The effect of glycaemic events (both hypoglycaemia and hyperglycaemia) on the ECG signals across different times of the day will be examined. Before performance of data analytical methods, we aim to deploy data pre-processing methods to extract relevant patterns from raw ECG signals. The extracted ECG signals may be affected by noise occurred due to body movement, respiration, and device electrodes. Therefore, we will perform baseline wander removal [40, 41] in order to remove low frequency noise followed by signal normalization at zero mean and unit variance. We will then perform the ECG segmentation in order to segment ECG beats and detect fiducial points using our segmentation tool developed in a recent study for cardiovascular disease detection [42]. We will divide the ECG data into five to fifteen minutes excerpts in order to annotate them as normal, severe hypoglycaemic or severe hyperglycaemic events [43]. Population will be described in terms of demographic and clinical characteristics. For CGM signals, considering the different frequencies of glucose readings across the devices (Table 2), we perform linear interpolation to estimate blood glucose measurements at every second with respect to real time ECG signals.

#### Data analysis

Processed data will be analysed using statistical software (e.g., SPSS) and programming languages (such as Python or R) using descriptive statistics and inferential statistics. We aim to determine HRV parameters using the fiducial points determined from our ECG segmentation tool. The HRV parameters can be categorized into time-domain features, frequency domain features and non-linear features [31]. Time-domain features include the length and slope among the fiducial points (P, Q, R, S and T). The frequency-domain features include power spectrum in different frequency bands of the short-term ECG excerpts. Other nonlinear features include fluctuations and entropy of ECG excerpts. We will use the packages from Python such as neurokit2 [44] and heartpy [45] in order to determine frequency-domain features and other nonlinear features. We will perform statistical tests such as ANOVA, Mann-Whitney U Test and Wilcoxon signed tests to determine the statistical significant difference among HRV features from different glycaemic events [46, 47].

#### Data modelling

The inherent part of the project is to develop the mathematical models in order to predict and detect the glycaemic events based on ECG signals. The mathematical modelling will lead to development of the artificial intelligence-based models. The artificial intelligence based models vary from traditional machine learning models (such as Support Vector Machines (SVM), Decision Trees, Artificial Neural Networks (ANN) etc.) to advanced deep learning based models [48]. The traditional machine learning models have the capability to train the static features extracted in both time and frequency domains. However, before training the model, we need to apply a features selection step in order to determine the most suitable features relevant to the glycaemic event classification [49]. On the other hand, deep learning models have the capability to train ECG beat features along with their temporal context without prior requirement of feature selection methods [50]. For modelling purposes, we aim to use well-known frameworks and packages such as tensorflow, theano, keras, torch, spark, pandas, scikit-learn, matlab etc. [51].

### Ethics approval

The study protocol adheres to the ethical principles stated in the Declaration of Helsinki and the National Health and Medical Research Council statement and was approved by the Ethical Committee of Bambino Gesù Children’s Hospital, Rome, Italy (2260_OPBG_2020). All participants and their parents or caregivers will be provided with complete information related to their future participation to the study. They will be required to fill in a written consent form, where the methods, advantages and disadvantages in taking part to the experiment will be explained. This will provide a clear understanding of the procedure, assure that their information will be kept strictly confidential, that their participation is voluntary, and that they have a right to withdraw at any time during the study. This study participation will not impose any risks for patients and will not affect in any way the clinical path of the recruited patients, as they will continue to receive their usual diabetes care, and they will continue to manage diabetes as usual.

## Results

Data collection is expected to be completed approximately by June 2023. It is expected that sufficient data will be collected to develop and validate the generalized AI algorithm. Study results will be disseminated through peer reviewed journals, a doctoral thesis and conference presentations.

## Discussion

The main purpose of this study is the development of automated methods for non-invasive detection of hypoglycaemic and hyperglycaemic events among paediatric patients suffering from Type 1 Diabetes (T1D). Although glycaemic events can result in acute complications among children and adolescent more frequently than in adults, however their effective management can be helpful in avoiding chronic conditions that could also result in death. Under current clinical setup, the glycaemic events are determined using invasive and cumbersome finger prick methods, which can lead the paediatric population not to be fully compliant with the diabetic management protocols. Therefore, the development of non-invasive methods is pivotal for optimal T1D management among paediatric population.

The primary aim of this observational study is to validate and improve a non-invasive method for the detection of blood glucose levels, developed during a pilot work in 2020 [43]. This pilot work was based on detection of hypoglycaemic events based on ECG signals from healthy subjects. Results of the pilot study showed that the developed personalized classifiers based on deep-learning artificial intelligence algorithms, can perform automatic detection of hypoglycaemic events using the morphology and the short-term ECG intervals recorded with wearable devices in free-living conditions. This study is designed to improve the efficiency and robustness of the personalized model towards Type 1 Diabetes patients.

## Conclusion

This study approved by the Ethical Committee on March 2021 (2260_OPBG_2020). Recruitment started on May 2021 and is expected to be completed approximately by June 2023.

The primary aim of this study is the development of the generalized AI model for detection of hypoglycaemic and hyperglycaemic events in both healthy and diabetic patients. The generalized model needs to be trained and tested in a variety of healthy and diabetic subjects for accurate detection of glycaemic events. The development of the accurate and robust generalized model is pivotal for efficient management of the diabetes in paediatric population, drastically reducing pain and discomfort of using invasive methods to continuously measure glucose levels.

As secondary outcomes, this study aims at investigating clinical variables and quality of life of patients recruited. These variables will be investigated in order to provide a database for paediatric patients. Publicly available data on paediatric diabetes patients are still lacking, and this study will also aim at collecting clinical and quality of life data for this category of patients.

We believe that this intervention will strongly support and progress diabetes care research for children and young adults with type 1 diabetes, improving glycaemic control and consequently the quality of life.

## Electronic supplementary material

Below is the link to the electronic supplementary material.

Supplementary Material 1

## Footnotes

1 An Assessment of the Zephyr BioPatch and Its Ability to Monitor Patient Position | Smart Patients. https://www.smartpatients.com/trials/NCT02548624., Performance Assessment of a Remote Patient Monitoring Device - ICH GCP - Clinical Trials Registry. https://ichgcp.net/clinical-trials-registry/NCT02570906.
